# Bacteriological Quality and Heavy Metal Analysis of Packaged Water Produced in Lusaka, Zambia and Associated Quality Control Measures

**DOI:** 10.3389/fpubh.2021.620700

**Published:** 2021-06-15

**Authors:** Rodney K. Banda, Patricia Mubita, Given Moonga, Chisala D. Meki

**Affiliations:** ^1^Department of Environmental Health, School of Public Health, University of Zambia, Lusaka, Zambia; ^2^Department of Epidemiology and Biostatistics, School of Public Health, University of Zambia, Lusaka, Zambia

**Keywords:** packaged water, quality control, bacteriological water quality, chemical water quality, lead concentration

## Abstract

Many people in the world lack safe basic drinking water sources and rely on untreated water source. Packaged water can be considered as an alternative to other water sources if measures are put in place to ensure its safety for consumption. This study aimed to assess the bacteriological quality and heavy metal analysis of packaged water produced in Lusaka, Zambia and associated quality control measures. A cross-sectional study was conducted in May 2019 where 18 brands of packaged water were analyzed for total and fecal coliforms as well as concentrations of Lead, Chromium, and Cadmium. The study found that 33.5% of the packaged water produced in Lusaka did not comply with the standard for drinking water on bacteriological quality. We also found that the concentrations for Lead were <0.01 mg/l in all the 17 samples, thus compliant to WHO/ZABS standards. Concentrations of Chromium were between 0.002 and 0.62 mg/l and compliance to the standard was 11.8%. Concentrations for Cadmium were between 0.009 and 0.2 mg/l against the acceptable concentration of <0.003 mg/l. Most brands of the packaged water did not conform to the standards for drinking water.

## Highlights

- Informs consumers and regulatory authorities on the quality of packaged water.
- Shows the concentrations of heavy metals in packaged water.
- Brings out successes and challenges in water quality control.
- Informs companies producing water on recommended best practices.
- Informs policy on areas of improvement in the water sector.

## Introduction

Water must be adequate, safe and accessible in order to support health and protect the public from ill health. Diseases related to contaminated water are a major burden on human health and improved quality of drinking-water provide significant health benefits (1). It is further reported that globally, at least 2 billion people use a drinking water source contaminated with feces with an estimation of 502, 000 deaths due to diarrhea each year (1). Due to the increasing demand for access to safe drinking water, the global population has turned to the use of packaged water (2). Packaged water is natural or treated water that reaches the consumer as a packaged product in either a glass or plastic container (3). Packaged water is produced by processing raw water from springs, rivers, or boreholes and can either be natural or purified. The quality of packaged water is dependent on quality control measures by companies producing packaged water and external monitoring agencies.

Choice of packaged water is influenced by social norms, safety and image on consumer choice. The ever-increasing popularity of packaged water means that it is of the utmost importance to determine not only their mineral content, but above all, the content of possible contaminants. A study in Dharan municipality in Nepal revealed presence of total coliforms in 25% of bottled water though none of the bottled water samples had fecal coliforms (4). In a similar study on the microbiological quality of sachet water sold in Maiduguri metropolis, Nigeria revealed the presence of *coliforms, E. coli, Pseudomonas* sp., and *Salmonella* sp. Ninety-five percent (95%) were not fit for human consumption (5). The study revealed that this could have been as a result of inadequate sanitation and unhygienic practices or ineffective or malfunctioning water treatment processes.

A study conducted in Pretoria South Africa found that two brands of eight brands of packaged water did not comply with the South African Bureau of Standards (SABS) guidelines for microbial quality of drinking water (6). It was further concluded that microbial contamination of packaged natural water was most likely to occur due to improperly cleaned equipment and bottles, failure of ozonation or ultraviolet (UV) equipment or due to contamination of the water by workers. In a related study conducted by Meki et al. (7) on the bacteriological quality of bottled water sold in Lusaka district, Zambia. It was revealed that 8.9% of the sampled water did not meet Zambia Bureau of Standards (ZABS) and World Health Organization (WHO) bacteriological standards for drinking water quality. Packaged water production has been a growing industry in Zambia and most people in Zambia perceive packaged water to be better than tap water in terms of quality (8). Unsafe drinking water can lead to the spread of water borne diseases. The recent Cholera outbreak in Zambia which started on 6th October 2017 and ended on 18th May 2018 recorded 5, 905 suspected cases of Cholera in the 10 provinces of Zambia (9). Of these suspected cases, 5,414 (91.7%) were recorded in Lusaka with 98 deaths representing a case fatality rate of 1.8% (9). The outbreak was partly attributed to unsafe drinking water. This occurrence shows that although packaged water is considered one of the safe water sources, it can cause water borne diseases if unsafe.

Drinking water may also contain heavy metals in concentrations above which renders the water unfit for consumption. Heavy metals are elements with a weight 4–5 times as much as the weight of water (10) Heavy metals such as lead, chromium, and cadmium are dangerous in man because of toxicity and biological accumulation (10). The maximum acceptable level of lead, chromium, and cadmium are 0.01, 0.05, and 0.003 mg/l, respectively (11, 12). Higher concentrations of heavy metals cause physiological effects on the digestive, circulatory, nervous and other body systems (13). Lead and Cadmium are potent neurotoxins that accumulate in soft tissues and bone over time and are carcinogenic.

A study in Liaoning Province in China documented the increased risk of stomach cancer following ingestion of chromium in drinking water (14). Contamination of drinking water with heavy metals occurs due to both geogenic and anthropogenic activities. The geogenic activities include weathering and erosion of bedrocks; ore deposits; and volcanic eruptions. Anthropogenic activities that may cause contamination include agriculture, mining, industrial effluent, irrigation and solid waste disposal.

A similar study on the microbial and physicochemical quality of packaged water produced in Hamadan province of Iran revealed that all measured parameters of packaged water in Hamadan province were within acceptable range of the national and international standards (2). Chromium and Cadmium have similar effects but there is scanty information on their occurrence in packaged drinking water in Zambia.

A study on the extent to which drinking water is tested for compliance in sub-Saharan Africa including Zambia revealed that regulated water quality monitoring activities did not achieve testing levels specified by WHO Guidelines or national standards, particularly among smaller water suppliers and surveillance agencies (15). The analysis showed that bureaucratic procedures such as the development of national standards and the creation of independent regulatory bodies are unlikely to solve the problem without parallel commitments to implementation of the policies which include greater resource allocations for monitoring in small towns and focusing on improving the cost-effectiveness of water quality monitoring. Cost-effectiveness of risk-management procedures include applying sanitary surveys to reduce testing of clearly contaminated supplies and prioritizing water quality parameters that represent the greatest risks to public health. Coordination between surveillance agencies and private sector equipment suppliers would improve the supply of testing equipment and consumables. Furthermore, institutions require the resources and skills to act upon testing results to improve water quality. Finally, capacity building of monitoring programs should focus on program sustainability and applying water quality data toward improved water safety.

In Zambia, water quality monitoring is conducted by authorities that include local authorities, Ministry of Health, Zambia Compulsory Standards Agency (ZCSA) and the Competition and Consumer Protection Commission (CCPC). This study aimed to assess the bacteriological quality and heavy metal analysis of packaged water produced in Lusaka and associated quality control measures. Findings revealed that most of the brands of packaged water did not comply with the ZABS/WHO standards for drinking water.

## Materials and Methods

An analytical cross-sectional study design was conducted in May 2019 to answer the research questions. The quality of packaged water and quality control measures were assessed at the same time. The study was conducted in the capital city of Zambia, which is Lusaka located by coordinates; 15°25′ South of the equator and 28°17′ east of Greenwich. The city has a population of 2,566,758. Lusaka is one of the fastest growing cities in Zambia. It experienced rapid urban growth of about 23% increase in the total urban area from 1990 to 2010 (16). Being a center of commerce, new businesses including water bottling have been emerging (16). Companies producing package water are located in industrial area or areas in close proximity to the industrial area. Groundwater sources within the area are used as the major sources of the packaged water. The study population consisted of all companies producing packaged water in Lusaka.

There were a total of forty-six (46) companies producing packaged water in Lusaka. The study also included Lusaka City Council (LCC) and ZCSA Companies which were closed during the study and those which did not consent to be part of the study were excluded. Total enumeration of the forty-six (46) companies producing water in Lusaka was conducted. Nine (9) companies were not operational, six (6) were outside Lusaka District, seven (7) were not traced and seven (7) did not consent to be part of the study leaving seventeen (17) companies which were included in the sample.

The study had two dependent variables: Bacteriological quality of packaged water and chemical quality of packaged water. Indicators for the bacteriological quality were total coliforms (absent in 100 ml of water present in 100 ml of water) and fecal coliforms (absent in 100 ml of water; present in 100 ml of water). Heavy metal analysis had three indicators: lead (Less than 0.01 mg/l and more than 0.01 mg/l); cadmium (less than 0.003 mg/l and more than 0.003 mg/l); and chromium (less than 0.05 ml/l and more than 0.05 ml/l).

Independent variables included Zambia Bureau of Standards registration (registered unregistered); License from LCC (licensed or unlicensed); category of packaged water (natural or purified); abstraction source of the water intended for packaging (borehole, deep well, spring, or water utility); presence of functional chemical laboratory (presence = 0, absence = 1); frequency of heavy metal analysis (once a week, once a month, once every 3 months or once every 6 months) and; heavy metal removal during processing (being done or not done). Other independent variables were frequency of inspections and water testing by LCC (once every 3 months or once every 6 months); frequency of inspections and water testing by ZCSA (once every 3 months, once every 6 months); evidence of a functional bacteriology laboratory (present or absent); and frequency of bacteriological water testing (after each batch, once a week, once a month, every 3 months or every 6 months);

Data was collected by the Principal Investigator and two trained Research Assistants. Water samples from the companies were collected from batches ready for distribution. Simple random sampling was used to collect the water samples by conducting a raffle for the last 20 cases of water produced. Pieces of paper with numbers 1–20 were put in a bag and the researcher picked a number representing the case where the bottles were to be picked. One bottle or sachet of a minimum of 500 ml capacity for each brand was collected for bacteriological analysis. A minimum of 1 l was required for chemical analysis according to the ZABS guidelines. For some brands of water that were packaged in packages of <1 l, the researcher had to combine two or three bottles or packages to come up with the required quantity of the water. The samples were kept in a cooler box packed with ice blocks at temperatures from 4 to 10°C and transported to the ZABS laboratory in Lusaka within 24 h of collection for analysis. This was to make sure that the microorganisms that may have been present did not grow and multiply.

Membrane filtration method was used for bacteriological analysis of the water because it allows for isolation and enumeration of discrete colonies of bacteria. After receiving the sample at the laboratory, necessary dilutions were made. The absorbent pad was added to the Petri dish. The pad was soaked with Lactose agar with Tergitol 7. Flamed forceps were used to remove the membrane filter from the sterile package and was placed into the filtration apparatus. The water sample was then added to the filtration apparatus and a vacuum was applied to the suction flask. The filter was then removed with sterile forceps from the funnel and placed in the prepared Petri dish and incubated at 36°C for 24 h for total coliforms and 44.5°C for fecal coliforms. The colonies were then counted and reported. The term too numerous to count was used to denote colonies which were more than 200 in number. The laboratory used TEST-1-0013 of the ISO/IEC 17025 SADCAS accredited method to analyze the samples. Measures done to assure quality of the results included replicate testing and replicate evaluation of test results.

Analyses for lead, cadmium and chromium were conducted by atomic absorption spectroscopy (AAS) by an atomic absorption spectrophotometer model number AA-6800. The laboratory used TEST-8 0018 of the ISO/IEC 17025 SADCAS accredited method to analyze the samples. Quality control was assured by calibrating the equipment using an approved caliberant; checking digestion efficiency and system performance; and adhering to internal standards.

Observational checklists were used for observations and document reviews for quality control measures. Data on the quality control measures was collected from the companies, LCC and ZCSA. A senior member of the production staff was interviewed using a structured questionnaire frequency of inspection. A checklist was also used for observations and document review to verify the information. A senior member in the health inspectorate section was interviewed at LCC as well as a senior member of staff at ZCSA using a questionnaire.

Stata software version 15 was used for data entry and analysis. Frequencies and proportions were used to report descriptive statistics for bacteriological quality and quality control measures implemented by companies. Means and ranges were used to describe the concentrations for Lead, Chromium, and Cadmium in comparison with the WHO/ZABS standards. The Fisher's exact tests were performed to establish associations between the bacteriological quality of packaged water and quality control measures as well as the heavy metals in packaged water and quality control measures. Analyses were done at 0.05 significance level.

Ethical clearance was sought from the University of Zambia's Biomedical Research Ethics Committee reference number 026-08-18. Thereafter, the researcher obtained permission from LCC, ZCSA and the companies. Lastly the researcher got written consent from companies which were part of the study. It was noted that the research findings may result in loss of business if the identity of the sampled companies were revealed. Therefore, anonymity was maintained throughout the study. Companies were not coerced into being part of the research. Companies were also advised on measures to take to maintain good internal quality control measures during data collection.

## Results

### Characteristics of Companies

Table 1 shows the characteristics of companies producing packaged water in Lusaka. Out of the 17 companies included in the study, the majority, 13 (76.5%) were licensed by LCC. Most companies, 16 (94.1%) were registered by the Zambia Bureau of Standards. Only 6 (35.3%) were inspected at least each quarter by LCC and only 4 (23.5%) were inspected at least every quarter by the Zambia Compulsory Standards Agency.

**Table 1:** Descriptive characteristics of companies that produce packaged water in Lusaka.

**Variable measure**		**Frequency (*n*)**	**Proportion (%)**
Licensing by LCC	Licensed	13	76.5
	Not licensed	4	23.5
Registered by ZABS	Registered	16	94.1
	Not registered	1	5.9
Frequency of inspection by LCC	Inspected	6	35.3
	Not inspected	11	64.7
Frequency of inspection by ZCSA	Inspected	4	23.5
	Not inspected	13	76.5
Abstraction Source	Spring	1	5.9
	Borehole	16	94.1
Category of mineral water	Natural mineral water	1	5.9
	Purified water	16	94.1
Bacteriology laboratory	Present	11	66.4
	Absent	6	35.3
Bacteriological testing of each batch	Tested	9	47.4
	Not tested	8	47.1
Laboratory for heavy metal analysis	Present	0	0
	Absent	17	100
Heavy metal extraction during processing	Done	0	0
	Not done	17	100

Almost all companies 16 (94.1%) used boreholes as abstraction sources for the packaged water. Only 1 (5.9%) used a spring as the source of the water. Only one company produced natural mineral water while the rest 16 (94.1%) produced Purified Mineral Water. Most companies 11 (66.4%) had laboratories for bacteriological analysis but only 9 (47.4%) conducted bacteriological testing of water samples for each batch produced. None of the companies had laboratories for heavy metal analysis nor did have means of extracting heavy metals from the water during processing.

### Bacteriological Quality

Table 2 shows the bacteriological quality of packaged water for the seventeen (17) companies. Of the 6 (35.3%) samples which had presence of total coliforms. None of the samples had presence of fecal coliforms. Compliance to bacteriological standards for drinking water was 64.7%.

**Table 2:** Bacteriological quality of packaged water in Lusaka.

**Water source**	**Total coliforms**			**Feacal coliforms**		
	**Present (%)**	**Absent (%)**	**Total (%)**	**Present (%)**	**Absent (%)**	**Total (%)**
Borehole	6 (35.3%)	10 (58.8%)	16 (94.1%)	0 (0.00%)	16 (94.1%)	16 (94.1%)
Spring	0 (0.00%)	1 (5.9%)	1 (5.9%)	0 (0.00%)	1 (5.9%)	1 (5.9%)
Total	6 (35.3%)	11 (64.7%)	17 (100%)	0 (0.00%)	17 (100%)	17 (100%)

### Concentrations of Lead, Chromium, and Cadmium in Packaged Water

Table 3 indicates laboratory results for chemical quality of the water. All the brands of packaged water were not compliant to the ZABS/WHO standards of drinking water as regard to levels of Chromium and Cadmium whereas the levels of lead were within acceptable limits.

**Table 3:** Concentrations of lead, chromium, and cadmium in packaged water.

**Water source**	**Lead**			**Chromium**			**Cadmium**		
	**% Satisfactory (<0.001)**	**% Unsatisfactory (>0.001)**	**Total**	**% Satisfactory (<0.05)**	**% Unsatisfactory (>0.05)**	**Total**	**% Satisfactory (<0.003)**	**% Unsatisfactory (>0.003)**	**Total**
Borehole	16 (94.1%)	0 (0.0%)	16 (94.1%)	2 (11.8%)	14 (82.4%)	16 (94.1%)	0 (0.00%)	16 (94.1%)	16 (94.1%)
Spring	1 (5.9%)	0 (0.0%)	1 (5.9%)	0 (0.0%)	1 (5.9%)	1 (5.6%)	0 (0.00%)	1 (5.9%)	1 (5.9%)
Total	17 (100%)	0 (0.0%)	17 (100%)	2 (11.8%)	15 (88.2%)	17 (100%)	0 (0.00%)	17 (100%)	17 (100%)

### Summary for Concentrations of Lead, Chromium, and Cadmium

Table 4 illustrates a summary of the concentrations for Lead, Chromium, and Cadmium. The concentrations for Lead were <0.01 mg/l in all the 17 samples giving a compliance of 100% to standards for drinking water quality. Concentrations for Chromium were as low as 0.002 mg/l and as high as 0.62 mg/l giving a compliance of 11.8% from the borehole source. Concentrations for Cadmium were as low as 0.009 mg/l and as high as 0.2 mg/l giving a compliance of 0%.

**Table 4:** Concentrations of lead, chromium, and cadmium.

**Element (mg/l)**	**Permitted**	**Mean**	**Std. Dev**.	**Min**	**Max**	**Compliance**
Lead	<0.01	<0.001	0	<0.001	<0.001	100%
Chromium	<0.05	0.2658	0.0443	0.002	0.62	11.8%
Cadmium	<0.003	0.0264	0.0109	0.009	0.2	0%

### Quality Control Measures Associated With Quality of Packaged Water

Table 5 shows the quality control measures associated with the bacteriological quality of package water. About half (41.7%) of the packaged water produced by companies with Health Permits from the local authority were satisfactory compared to 3 (17.66%) which were satisfactory but from companies without Health Permits. Only 1 (5.9%) of the packaged water from companies inspected quarterly by the local authority was unsatisfactory compared to 5 (29.5%) from companies which were not inspected every quarter. Packaged water produced by companies that own bacteriological laboratories accounted for 8 (47.1%) of satisfactory results while only 3 (17.3%) were satisfactory from companies without bacteriological laboratories. Companies that conducted bacteriological analysis on each batch accounted for 8 (47.1%) of satisfactory results while companies that did not conduct bacteriological analysis only accounted for 1 (5.9%) of satisfactory results.

**Table 5:** Quality control measures associated with bacteriological quality.

**Quality control measure**		**Bacteriological quality**		**Fisher's exact**
		**Satisfactory**	**Unsatisfactory**	***P*-values**
LCC health permit	Present	8 (47.1%)	4 (23.5%)	1.0
	Absent	3 (17.6%)	2 (11.8%)	
Inspection each quarter by LCC	Inspected	4 (23.5%)	1 (5.9%)	0.6
	Not inspected	7 (41.2%)	5 (29.5%)	
Inspection each quarter by ZCSA	Inspected	3 (17.6%)	0	0.5
	Not inspected	8 (47.1%)	6 (35.3%)	
ZABS certificate	Present	11 (64.7%)	5 (29.4%)	0.3
	Absent	0	1 (5.9%)	
Abstraction source	Spring	1 (5.9%)	0	1.0
	Borehole	10 (58.8%)	6 (35.3%)	
Bacteriological laboratory	Present	8 (47.1%)	3 (17.6%)	0.6
	Absent	3 (17.3%)	3 (17.6%)	
Bacteriological testing after each batch	Tested	8 (47.1%)	3 (17.6%)	0.05
	Not tested	1 (5.9%)	5 (29.4%)	

The Fisher's exact test revealed that only bacteriological testing after each batch was statistically significant a *p*-value of 0.05.

## Discussion

### Bacteriological Quality

The study revealed that 35.3% of the packaged water produced in Lusaka did not comply with the standards for drinking water.

The WHO and ZABS require that treated water for drinking purposes must have zero total coliforms in 100 ml of water as well as zero fecal coliforms in 100 ml of water. A lower compliance proportion was reported in a similar study where 8.9% of bottled water sold in Lusaka was not compliant with the standard for drinking water (7). The difference in compliance level could be because the current study focused on water produced in Lusaka while the 2014 study focused on water sold in Lusaka which may have included water produced from other districts.

The total coliforms detected in the water may have been due to inadequate treatment. This also highlights the inability of companies to use methods that can adequately treat the water. Water treatment methods are meant to make the water safe but this was not the case in some companies. For example, three brands had more than 200 total coliforms which were recorded as too numerous to count. The presence of total coliforms does not imply fecal contamination but indicates other sources of contamination that should be analyzed to decide the route the organisms are entering the water system (17). The risk of contracting a water-borne illness is increased with the water tests positives for coliforms.

The 35.3% non-compliance with the bacteriological quality of drinking water is a serious public health concern as it could lead to diarrheal diseases. Zambia had a serious Cholera outbreak in 2017 which resulted in about 5, 900 cases and 114 deaths a majority of which was recorded in Lusaka (9). People perceive packaged water as safe but it could contribute to such outbreaks if quality control measures are not implemented well.

### Heavy Metal Concentrations in Packaged Water

Heavy metal concentrations in packaged water depend on many factors that include mineralogy of rocks encountered during abstraction, residence time of groundwater in the aquifer and topology ((18)). This study revealed that the concentrations of Lead were below 0.01 mg/l in all the brands of the packaged water. The compliance on the concentration of lead could be attributed to the geology of the area. The underlying rock may not contain Lead therefore the water abstracted show low lead concentrations.

Only 11.8% of the brands had Chromium concentrations <0.05 mg/l. Chronic exposure to chromium has been linked to cancer and other non- carcinogenic health effects such as cardiovascular disease, neurological deficits and hypertension (19). Chromium targets the iron- and 2-oxoglutarate-dependent dioxygenase family enzymes and other histone modifying enzymes to mediate the toxicity and carcinogenicity (19).

None of the brands had Cadmium concentrations below 0.003 mg/l. This could be attributed to natural or anthropogenic activities. The aquifer could contain high levels of Chromium and Cadmium due to weathering and erosion of bedrocks or ore deposits. The high concentrations of the heavy metals could also be due to anthropogenic activities that include agriculture; mining; discharge of industrial effluent from industries; irrigation using wastewater irrigation and solid waste disposal. It is also important to note that all the companies in this study were located in the industrial area.

The underlying geology, soil type, and soil thickness in Lusaka makes the ground water vulnerable to contamination from industrial effluent (20). No further treatment is conducted by the companies to bring the levels to acceptable levels.

High concentrations of Cadmium can cause high blood pressure and destroy red blood cells and testicular tissue (21). Cadmium also has the potential to cause health effect that include vomiting, diarrhea, muscle cramps, sensory disturbances, liver injury, convulsions, shock, and renal failure in a short period of time (22). Exposure for a long period of time at levels exceeding 0.05 mg/l can cause kidney, liver and bone damage (11).

About 50% of accumulate dose of Cadmium is stored in the kidneys and cause tubular injury which leads to tubular dysfunction with urinary loss of glucose and amino acids as well as bicarbonate and phosphates (23). The loss of vitamin D binding protein in urine also indirectly contribute to osteomalacia in adults and rickets in children.

All three analyses were conducted on each sample implying that the overall compliance in respect of the three heavy metals under the study was 0%.

All companies used groundwater as the abstraction source for the water. The season of the year could have also contributed to the observed concentrations as the water levels may be low thereby increasing the concentrations of the elements. The geology of the study area could contain rocks with chromium and cadmium and these are extracted together with the water. Furthermore, all the companies are located in industrial area or areas in close proximity to the industrial area. Boreholes and the spring for companies producing purified and natural mineral water, respectively, are located in the same area. The pollution of ground water may be from the geology of the areal or industrial effluent.

Since water is taken daily with most people taking at least 1 l a day, heavy metal in drinking water can bio accumulate and may cause health problems in time. It is important to note that Chromium and Cadmium are neural toxins which results to birth defects (11).

### Quality Control Measures

Quality control measures for production of packaged water are done internally by the companies and externally by government agencies. Licensing and registration by LCC and the ZABS, respectively, is dependent on compliance to set standards. For example, compliance to hygiene standards at all times is one of the conditions for issuance of the Health Permit from the local authority.

Notably, companies which implemented the quality control measures considered in the study had most of the brands complying with the standards for drinking water. About half of the packaged water produced by companies with Health Permits from the local authority were satisfactory compared to only 17.6% which were satisfactory but from companies without Health Permits. Good hygiene is one of the requirements before issuance of the health permit therefore companies with Health Permits are less likely to have sources of contamination. In addition, the frequency of inspections also plays a part in the maintenance of hygiene as can be explained from the 5.9% of the packaged water from companies inspected quarterly by the local authority which was unsatisfactory compared to 29.5% from companies which are not inspected every quarter.

Presence of a bacteriological laboratory also plays a part as companies become aware of areas of improvement in terms of preventing contamination. Packaged water produced by companies that owned bacteriological laboratories accounted for 47.1% of satisfactory results compared to only 17.3% from companies without bacteriological laboratories. Furthermore, conducting bacteriological analysis on each batch that is produced influences the quality of the water as companies are able to isolate unfit batches.

Only bacteriological testing after each batch was found statistically significant. The other factors many not have been statistically significant because of the small sample size. However, the explanation given above gives an insight of how they are associated with the bacteriological quality of the packaged water.

All brands had concentrations of Lead within acceptable concentration of <0.01 mg/l. On the other hand, none of the brands were compliant to the chemical quality on Cadmium concentrations and only 11.8% were complaint on Chromium concentrations. None of the companies use methods or technologies to extract the heavy metals during processing of the water. This finding confirms a 2016 study which revealed that many developing countries have a challenge of reducing human exposure to heavy metals, due to inadequate financial capacities to use advanced technologies for heavy metal removal (24). Accessibility of these technologies coupled with weak monitoring mechanisms may also exacerbate the problem.

The focus by companies and government agencies has been on measures to ensure acceptable bacteriological quality of the water. Measures to ensure that only sources that do not contain heavy metals are used or measures to remove heavy metals from the water during processing are not taken by both the government agencies and the companies.

### Limitations, Discussion of Methods, Bias and Validity of the Study

The study was a cross-sectional design which only provided data at single point in time. Collection of data over a long period of time would reveal more information of which months or season of the year the quality of water improves or becomes poor. Furthermore, samples were only analyzed at one laboratory without any confirmatory tests at other laboratories.

The data collection tools used in the current study were not validated and prone to affect the quality of the information acquired. To ensure internal validity, the questionnaires and checklists were pretested before use. The principal investigator collected the data himself with assistance of trained assistants. Triangulation of the data was used when collecting certain information by using checklists, document review and observations. For instance, data on frequency of water testing was done by interviews and document review and data on presence of laboratory was collected by interview and observation.

The companies which did not consent would have added value to the study had they participated in the study. Some companies were also not traced during the study because the information on the labels applied on the packages was inadequate or missing. For instance some labels had no physical addresses and some only had postal addresses. This raises the concern of authenticity of the companies on whether they really exist or it is a way of hiding from authorities.

Despite the limitations, the study is valid and can be generalized because of the complete enumeration of companies producing packaged water and random sampling of the water samples.

## Conclusions

The study revealed that 35.3% of packaged drinking water did not comply with the WHO/ZABS standards for drinking water quality of zero total or fecal coliforms in 100 ml of water. All brands of packaged water had concentrations of Lead within acceptable concentration of <0.01 mg/l. On the other hand, none of the brands were compliant to the chemical quality on concentration of Cadmium and only 11.8% were satisfactory on the concentration of Chromium. However, the analyses for the three heavy metals were done on each brand and therefore none of the packaged water brands met the standards for chemical quality of drinking water. Quality control measures associated with the quality of packaged water produced in Lusaka are inadequate as can be seen from the quality of the water. However, measures to ensure bacteriological quality are better implemented than measures meant to ensure chemical quality. Companies and government agencies need to ensure that the packaged water produced is safe for consumption. Only safe sources should be used for abstraction of water for packaging. However, if the aquifer is naturally unsafe, adequate measures need to be put in place by companies to remove heavy metals to ensure that the final product is safe.

## Data Availability Statement

The raw data supporting the conclusions of this article will be made available by the authors, without undue reservation.

## Ethics Statement

The studies involving human participants were reviewed and approved by the University of Zambia's Biomedical Research Ethics Committee (reference number 026-08-18). The participants provided their written informed consent to participate in this study.

## Author Contributions

RB: conceptualization, methodology, formal analysis, investigation, resources, data curation, writing—original draft preparation, writing—review and editing, and visualization. CM, PM, and GM: supervision. All authors have read and agreed to the published version of the manuscript.

## Conflict of Interest

The authors declare that the research was conducted in the absence of any commercial or financial relationships that could be construed as a potential conflict of interest.
